# Potentially Biodynamic Tetraaza Macrocycles and their Manganese Complexes: Antiandrogen, Antimicrobial and PDI Studies

**DOI:** 10.1155/BCA.2005.161

**Published:** 2005

**Authors:** Ashu Chaudhary, Anita Phor, R. V. Singh

**Affiliations:** 1 Department of Chemistry University of Rajasthan Jaipur 302 004 India; 2 Hindu College Sonipat 131001 India

## Abstract

Fourteen to eighteen membered tetraazamacrocyclic ligands N_4_TTD^1^–N_4_TTD^4^ have been synthesized by the condensation of aliphatic diamines. H_2_N–(CH_2_)_y_–NH_2_ (y = 2 or 3) and dicarboxylic acids, HOOC-(CH_2_)_x_–COOH (x = 1 or 2) in the presence of condensing reagents dicyclohexylcarbodiimide (DCHC) and 4-dimethylaminopyridine (DMAP). On reduction these macrocyclic ligands give N_4_TTD^5^-N_4_TTD^8^, which form complexes with manganese(II) acetate. The new products with octahedral geometry have been characterized by elemental analyses, molecular weight determinations, magnetic moment and spectral studies *viz*., infrared, electronic, mass and X-ray. On the basis of the spectral studies the binding sites are proposed as the nitrogen atom of the macrocycles. The formulation of the complexes as [Mn(CH_3_COO)_2_(N_4_TTD^n^)] (where n = 1 - 8) has been established on the basis of chemical composition. To assess the growth inhibiting potential of the ligands and their manganese (II) complexes biological screening have been undertaken. The testicular morphology, testicular sperm density, sperm motility, density of cauda epididymal spermatozoa and fertility in mating trials and biochemical parameters of reproductive organs with ligands and their corresponding complexes, in vivo have also been described in the this communication.


### Potentially Biodynamic Tetraaza Macrocycles and their Manganese Complexes : Antiandrogen, Antimicrobial and PDI Studies

Ashu Chaudhary[1], Anita Phor[2] and R.V. Singh[1*]

[1]*Department of Chemistry, University of Rajasthan, Jaipur-302 004, India*
[2]*Hindu College Sonipat – 131001, India*

### ABSTRACT

Fourteen to eighteen membered tetraazamacrocyclic ligands $N_4TTD^1$–$N_4TTD^4$ have been synthesized by the condensation of aliphatic diamines $H_2N–(CH_2)_y–NH_2$ (y = 2 or 3) and dicarboxylic acids, HOOC–$(CH_2)_x$–COOH (x = 1 or 2) in the presence of condensing reagents dicyclohexylcarbodiimide (DCHC) and 4-dimethylaminopyridine (DMAP). On reduction these macrocyclic ligands give $N_4TTD^5$-$N_4TTD^8$, which form complexes with manganese(II) acetate. The new products with octahedral geometry have been characterized by elemental analyses, molecular weight determinations, magnetic moment and spectral studies *viz.*, infrared, electronic, mass and X-ray. On the basis of the spectral studies the binding sites are proposed as the nitrogen atom of the macrocycles. The formulation of the complexes as $[Mn(CH_3COO)_2(N_4TTD^n)]$ (where n = 1 - 8) has been established on the basis of chemical composition. To assess the growth inhibiting potential of the ligands and their manganese (II) complexes biological screening have been undertaken. The testicular morphology, testicular sperm density, sperm motility, density of cauda epididymal spermatozoa and fertility in mating trials and biochemical parameters of reproductive organs with ligands and their corresponding complexes, in vivo have also been described in the this communication.

### INTRODUCTION

The multifarious roles of transition metals in biochemistry /1-5/ suggested that consideration potential exists for the development of new chemistries with these metals in ligand systems specifically designed to serve these roles. The enormous interest in the synthesis of the transition metal complexes of the nitrogen donor ligands arises due to the wide range of pharmacological activities of these compounds, which in several cases are known to have been enhanced by the presence of transition metals /6,7/. The synthesis of a series of tetraamide macrocycles which have been designed to afford strongly donating tetraamido-N ligands upon tetradeprotonation and to be resistant to oxidative degradation has been described. It is demonstrated by

* Corresponding author: Professor R.V. Singh, Department of Chemistry, University of Rajasthan, Jaipur-302004, India E-mail : kudiwal@datainfosys.net : Fax +91-141-2708621

the preparation and characterization of an unprecedented class of square-planar cobalt compounds with high positive formal reduction potentials that the macrocycles possess the rare property of being compatible with strongly oxidizing coordination environment /8/. The complexes of the metal ions are significant because of their resemblance with many natural systems, such as porphyrins and cobalamines. The biological activity of this class of compounds is associated with the chelation /7/. They are known to function as antimicrobial /9/, antifertility, antimalaria and antileukemic agents /10/. Many of these transition metal ions in living systems work as enzymes carriers in a macrocyclic ligand field environment.

Macrocyclic ligands display a number of features of chemical interest. Research into the synthesis, structure and properties of transition metal macrocyclic complexes to model a wide variety of metalloprotein active sites or to mimic their chemistry is well established /11/. Collins *et al.* /12/ have also described synthesis and characterization of the macrocyclic tetraamido-N-ligands. These tetraanionic ligands are strong donors and are resistant to the oxidative degradation. It has allowed rare high-valent metal centers such as five coordinated Fe (IV) and four coordinated Ni (III) to be isolated and fully characterized. Macrocycles have wide applications in medicine, cancer diagnosis /13/ and in the treatment of tumors /14/. Manganese chloride causes loss of testicular germ cells in rats and rabbits /15/ and decreased libido and impotency were also noted in a man occupationally exposed to manganese /16/. Macrocyclic complexes of Mn(II) exhibit a broad spectrum of biological activity. No work has been reported on the manganese(II) complexes with such type of tetraazamacrocyclic ligands. Therefore, the importance of the metal-nitrogen bonding and their prominence in agriculture, medicinal and industrial activity led us to synthesize and screen these compounds for their antifungal, antibacterial and antifertility activities.

### EXPERIMENTAL

All the glass apparatus used during the experimental work was fitted with quick fit interchangeable standard ground joints. The chemicals including dicyclohexylcarobodiimide, 4-dimethylaminopyridine, $Mn(CH_3COO)_2.4H_2O$, malonic acid, succinic acid (Fluka), amines and $LiAlH_4$ (E. Merck) were used as received.

### Synthesis of the Ligands ($N_4TTD^1$-$N_4TTD^4$)

The reaction was carried out in 2 : 2 molar ratios. The appropriate (1.5626g; 7.5 mmol) amount of dicyclohexylcarbodiimide and catalytic amount of 4-dimethylaminopyridine in 25 mL of dichloromethane at 0°C were kept magnetically stirred in a two-necked round bottom flask. The reaction was followed by the addition of 1,2-diaminoethane or 1,3-diaminopropane (corresponding to the dicyclohexylcarbo-diimide) in dichloromethane (25 mL) and malonic or succinic acid (corresponding to DCHC). The resulting reaction mixture was stirred for 10-12 hrs at 0°C. The solid product was isolated by filtration and washed with the same solvent and dried *in vacuo*. The solid products were recrystallized from benzene and dried *in vacuo*.

### Synthesis of the Ligands : ($N_4TTD^5$-$N_4TTD^8$)

The reaction was carried out in 1 : 2 molar ratio. The ligands $N_4TTD^1$-$N_4TTD^4$ (1.0g; 3.9 mmol) were dissolved in tetrahydrofuran (30 mL) and cooled at 0°C. Lithium aluminum hydride (corresponding to the ligands) in tetrahydrofuran (20 mL) was stirred for about 10 hrs in an ice bath. The reaction mixture was stirred under reflux for 72 hrs. After cooling it, 20 mL of water and 10 mL of 15% sodium hydroxide were added to the reaction mixture at 0°C. The solid material was filtered and the residue was washed with tetrahydrofuran. The filtrate and the tetrahydrofuran washings were concentrated under the reduced pressure.

### Synthesis of the Complexes

The reaction was carried out in 1 : 1 molar ratio. 0.9-0.8 g; 4.5-5.0 mmol ligands, $N_4TTD^5$-$N_4TTD^8$ were dissolved in methanol (50 mL). The reaction was followed by the addition of $Mn(CH_3COO)_2.4H_2O$ (corresponding to the ligands $N_4TTD^5$-$N_4TTD^8$) solution. The resulting mixture was stirred for 12 hours at 0°C. The solid product was filtered off and washed with the same solvent and dried in vacuo. The products were recrystallized from benzene. Thus a series of 14-18 membered tetraazamacrocyclic ligands and their complexes were derived by the condensation of dicarboxylic acids with primary diamines in the presence of condensing reagents DCHC and DMAP.

### Analytical Methods and Physical Measurements:

Conductivity measurements of $10^{-3}$M solutions were made with a Systronic Model 305 conductivity bridge in dry dimethylformamide at room temperature (28°C). Molecular weights were determined by the Rast Camphor Method. IR spectra were obtained as KBr pellets on a Perkin-Elmer 577 grating spectrophotometer in the range 4000-200 $cm^{-1}$ and far IR spectra were also recorded on the same spectrophotometer in Nujol Mulls using CsI cell. Electronic spectra in dimethylsulphoxide were recorded on a Hitachi W-2000 spectrophotometer. Magnetic moment of the complexes were determined by Gouy's method at room temperature. The mass spectrum was recorded on a Jeol SX 102/DA-6000 mass spectrometer/data system using Argon/Xenon (6 kV, 10mA). m-Nitrobenzyl alcohol (NBA) was used as the matrix. The accelerating voltage was 10 kV and the spectrum was recorded at the room temperature with the courtesy of the Central Drug Research Institute, Lucknow. The X-ray powder diffraction measurements were performed on the Philips P.W. 1840 automatic diffractometer using Fe(Kα) target with Mg filter. The wavelength used was 1.9373 Å and the reflection from 5.-65° was recorded. Manganese was estimated gravimetrically. Carbon and hydrogen analyses were performed at the Central Drug Research Institute, Lucknow.

### RESULTS AND DISCUSSION

The resulting new macrocyclic complexes are white or brown solids having sharp melting points. These are soluble in most of the organic solvents. The conductivity measured for $10^{-3}$ M solution in anhydrous DMF are in the range 9-29 $ohm^{-1}$ $cm^2$ $mol^{-1}$ showing them to be non-electrolytes. Elemental analyses agree well with the stoichiometry and chemical formulae of the compounds $[Mn(CH_3COO)_2(N_4TTD)^n]$. The molecular weight determinations indicated that all the complexes are monomers. The physical properties and analytical data of the complexes are given in Table 1.

**Table 1**

Physical Properties and Analytical Data of Tetraamide Ligands and their Corresponding Manganese(II) Complexes

| Compound | Empirical formula | M.P (°C) and Colour | Yield (%) | Analysis, Found (Calcd) % C | H | N | Mn | Mol.Wt Found (Calcd.) |
|---|---|---|---|---|---|---|---|---|
| $N_4TTD^1$ | $C_{10}H_{16}O_4N_4$ | 195 White | 73 | 46.80 (46.92) | 6.27 (6.28) | 20.77 (21.86) | - | 245 (256) |
| $N_4TTD^2$ | $C_{12}H_{20}O_4N_4$ | 209 White | 48 | 50.43 (50.57) | 7.04 (7.07) | 18.06 (19.66) | - | 251 (285) |
| $N_4TTD^3$ | $C_{12}H_{20}O_4N_4$ | 218 White | 38 | 50.44) (50.57) | 7.05 (7.05) | 18:41 (19.66) | - | 264 (285) |
| $N_4TTD^4$ | $C_{14}H_{24}O_4N_4$ | 202 White | 56 | 53.74 (53.90) | 7.52 (7.75) | 16.99 (17.96) | - | 299 (312) |
| $N_4TTD^5$ | $C_{10}H_{24}N_4$ | 173 White | 32 | 59.77 (60.90) | 11.88 (12.09) | 26.78 (28.01) | - | 181 (200) |
| $N_4TTD^6$ | $C_{12}H_{28}N_4$ | 154 Light brown | 35 | 63.21 (63.22) | 12.38 (12.32) | 23.44 (27.57) | - | 199 (228) |
| $N_4TTD^7$ | $C_{12}H_{28}N_4$ | 161 Light brown | 49 | 63.20 (63.22) | 12.19 (12.38) | 23.14 (24.57) | - | 211 (228) |
| $N_4TTD^8$ | $C_{14}H_{32}N_4$ | 168 Light brown | 52 | 65.53 (65.69) | 12.32 (12.60) | 20.99 (21.89) | - | 230 (256) |
| $[Mn(CH_3COO)_2(N_4TTD^5)]$ | $C_{14}H_{30}N_4O_4Mn$ | 209 Light brown | 27 | 44.94 (45.08) | 7.89 (8.10) | 15.02 (15.02) | 14.21 (14.72) | 345 (373) |
| $[Mn(CH_3COO)_2(N_4TTD^6)]$ | $C_{16}H_{34}N_4O_4Mn$ | 225 Light brown | 40 | 47.94 (47.77) | 7.80 (8.04) | 12.67 (13.97) | 13.11 (13.70) | 375 (401) |
| $[Mn(CH_3COO)_2(N_4TTD^7)]$ | $C_{16}H_{34}N_4O_4Mn$ | 218 Light brown | 34 | 47.81 47.78) | 7.76 (8.04) | 12.43 (13.97) | 13.61 (13.70) | 378 (401) |
| $[Mn(CH_3COO)_2(N_4TTD^8)]$ | $C_{18}H_{38}N_4O_4Mn$ | 211 Light brown | 31 | 50.11 (50.34) | 8.68 (8.92) | 12.09 (13.05) | 12.28 (12.80) | 397 (429) |

***Infrared Spectra :***

The first feature of all the complexes is the absence of $NH_2$ stretching vibrations of the amine and –OH groups of the dicarboxylic acids implying their involvement in the formulation of tetraamidemacrocycles. A single sharp band observed for the ligands $N_4TTD^1$-$N_4TTD^4$ in the region 3280-3292 $cm^{-1}$ may be assigned to ν(N-H) of amide group. The amide I, amide II, amide III, and amide IV groups are present at 1650-1675, 1540-1580, 1250-1260 and 630-645 $cm^{-1}$, respectively /17/. It provides a strong evidence for the presence of a closed cyclic product. Strong and sharp absorption bands appeared in the regions 2820-3055 and 1415-1475 $cm^{-1}$ in all the complexes are assigned to C-H stretching and C-H bending vibrational modes, respectively /18/. It has been noticed that tetraazamacrocycles $N_4TTD^5$-$N_4TTD^8$ do not show amide bands corresponding to the tetraamide macrocycles. However, a slight negative shift in the NH stretching vibration has been observed. None of the other bands show any appreciable changes.

In the spectra of the macrocyclic complexes $[Mn(CH_3COO)_2(N_4TTD^5)]$- $[Mn(CH_3COO)_2(N_4TTD^8)]$ as compared to their tetraazamacrocycles, the slight negative shift in the ν(N-H) band which appeared in the region 3200-3225 $cm^{-1}$ was noticed. It is ascribed to the coordinated N-H stretching vibration. This is further substantiated by the fact that all the complexes show a medium intensity band in the region 425-432 $cm^{-1}$ which is attributed to the Mn-N stretching vibrations /19/. The IR spectral data of the ligands and their corresponding manganese(II) complexes are listed in Table 2.

**Table 2**

IR Spectra Data (in $cm^{-1}$) of the Ligands and their Corresponding Manganese(II) Complexes.

| Compound | ν(N-H) | Amide | | | | C-H | | ν(Mn-N) |
|---|---|---|---|---|---|---|---|---|
| | | I | II | III | IV | Stretching | Bending | |
| $N_4TTD^1$ | 3290 | 1670 | 1540 | 1255 | 630 | 2843 | 1420 | - |
| $N_4TTD^2$ | 3284 | 1650 | 1568 | 1262 | 637 | 2820 | 1427 | - |
| $N_4TTD^3$ | 3288 | 1667 | 1580 | 1259 | 645 | 2836 | 1438 | - |
| $N_4TTD^4$ | 3283 | 1675 | 1577 | 1250 | 640 | 2854 | 1475 | - |
| $N_4TTD^5$ | 3286 | - | - | - | - | 2910 | 1466 | - |
| $N_4TTD^6$ | 3292 | | - | - | - | 2957 | 1470 | - |
| $N_4TTD^7$ | 3285 | - | - | - | - | 3037 | 1466 | - |
| $TTD^8$ | 3280 | - | - | - | - | 3020 | 1419 | - |
| $[Mn(CH_3COO)_2(N_4TTD^5)]$ | 3210 | - | - | - | - | 3041 | 1462 | 430 |
| $[Mn(CH_3COO)_2(N_4TTD^6)]$ | 3225 | - | - | - | - | 3055 | 1415 | 432 |
| $[Mn(CH_3COO)_2(N_4TTD^7)]$ | 3200 | - | - | - | - | 2970 | 1452 | 425 |
| $[Mn(CH_3COO)_2(N_4TTD^8)]$ | 3218 | - | - | - | - | 2990 | 1459 | 429 |

***Electronic Spectra***

The electronic spectra of the complexes $[Mn(CH_3COO)_2(N_4TTD^5)]$-$[Mn(CH_3COO)_2(N_4TTD^8)]$ display weak absorption bands in the regions 580-595, 420-435 and 380-386 nm for $^6A_{1g} \rightarrow {}^4T_{1g}$, $^6A_{1g} \rightarrow {}^4T_{2g}$ and $^6A_{1g} \rightarrow {}^4A_{1g}$, respectively. The values obtained correspond to these compounds reported earlier for the octahedral complexes /20/. The electronic spectral data of the ligands and their corresponding manganese(II) complexes are given in Table 3.

**Table 3**

Molar Conductance and Electronic Spectral Data of Tetraamide Ligands and their Corresponding Manganese(II) Complexes

| Compound | Molar conductance ($Ohm^{-1}$ $cm^2$ $mol^{-1}$) | Electronic spectral bands (nm) | | | Magnetic moment (B.M.) |
|---|---|---|---|---|---|
| | | $^6A_{1g} \rightarrow {}^4T_{1g}$ | $^6A_{1g} \rightarrow {}^4T_{2g}$ | $^6A_{1g} \rightarrow {}^4A_{1g}$ | |
| $[Mn(CH_3COO)_2(N_4TTD^5)]$ | 9 | 592 | 435 | 382 | 5.73 |
| $[Mn(CH_3COO)_2(N_4TTD^6)]$ | 17 | 580 | 432 | 380 | 5.78 |
| $[Mn(CH_3COO)_2(N_4TTD^7)]$ | 29 | 587 | 420 | 385 | 5.90 |
| $[Mn(CH_3COO)_2(N_4TTD^8)]$ | 20 | 595 | 428 | 386 | 5.80 |

***Magnetic Moment:***

The $\mu_B$ values for all the complexes are in the range 5.73-5.90 B.M. and suggest the high spin $d^5$ configuration for the complexes /21/.

***Mass Spectrum :***

The mass spectrum of the compound $[Mn(CH_3COO)_2(N_4TTD^8)]$ shows the molecular ion peak at m/z 430 $[M^+]$. The two acetate coordinated ions were removed with a mass loss of 118. The molecular cations during the fragmentation process obtained are shown in Fig.-1.

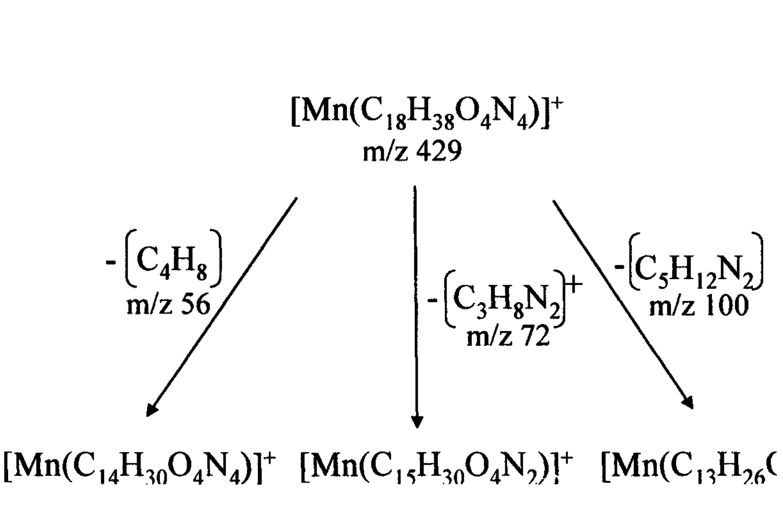

**Fig.1:** Mass Spectrum of the Compound [Mn($CH_3COO)_2(N_4TTD^8$)]

***X-ray Diffraction Studies :***

The X-ray diffraction analysis of the compound [Mn($CH_3COO)_2(N_4TTD^6$)] confirms the orthorhombic crystal system for this derivative having unit cell dimensions, a = 25.775, b = 17.542, c = 10.297 and $\alpha = \beta = \gamma = 90°$, Miller indices h, k and l are given in Table 4. The structure shown in Fig. 2 has been assigned to the complexes on the preceding spectral studies.

**Table 4**

X-ray Powder Diffraction Data of the Compound [Mn($CH_3COO)_2(N_4TTD^6$)]

| Peak no. | 2θ (Obs.) | 2θ (Calcd.) | Delta | h | k | l | δ-spacing (Obs.) Å |
|---|---|---|---|---|---|---|---|
| 1 | 15.36 | 15.35 | 0.00 | 2 | 2 | 0 | 7.250 |
| 2 | 16.91 | 16.89 | 0.02 | 3 | 0 | 1 | 6.590 |
| 3 | 22.62 | 22.59 | 0.03 | 5 | 1 | 0 | 4.940 |
| 4 | 25.85 | 25.85 | 0.00 | 4 | 3 | 0 | 4.330 |
| 5 | 27.85 | 27.47 | 0.00 | 5 | 2 | 1 | 4.080 |
| 6 | 30.78 | 30.76 | 0.02 | 3 | 4 | 1 | 3.650 |
| 7 | 31.95 | 31.91 | 0.04 | 3 | 3 | 2 | 3.520 |
| 8 | 37.94 | 37.91 | 0.03 | 7 | 3 | 1 | 2.980 |
| 9 | 42.21 | 42.22 | -0.00 | 4 | 3 | 3 | 2.690 |
| 10 | 44.65 | 44.65 | 0.00 | 10 | 1 | 0 | 2.550 |
| 11 | 46.38 | 46.38 | -0.00 | 2 | 7 | 0 | 2.460 |
| 12 | 48.25 | 48.18 | 0.07 | 8 | 5 | 0 | 2.37 |

Refined value of a = 25.775, b = 17.542, c = 10.297 (orthorhombic system) $\alpha = \beta = \gamma = 90°$

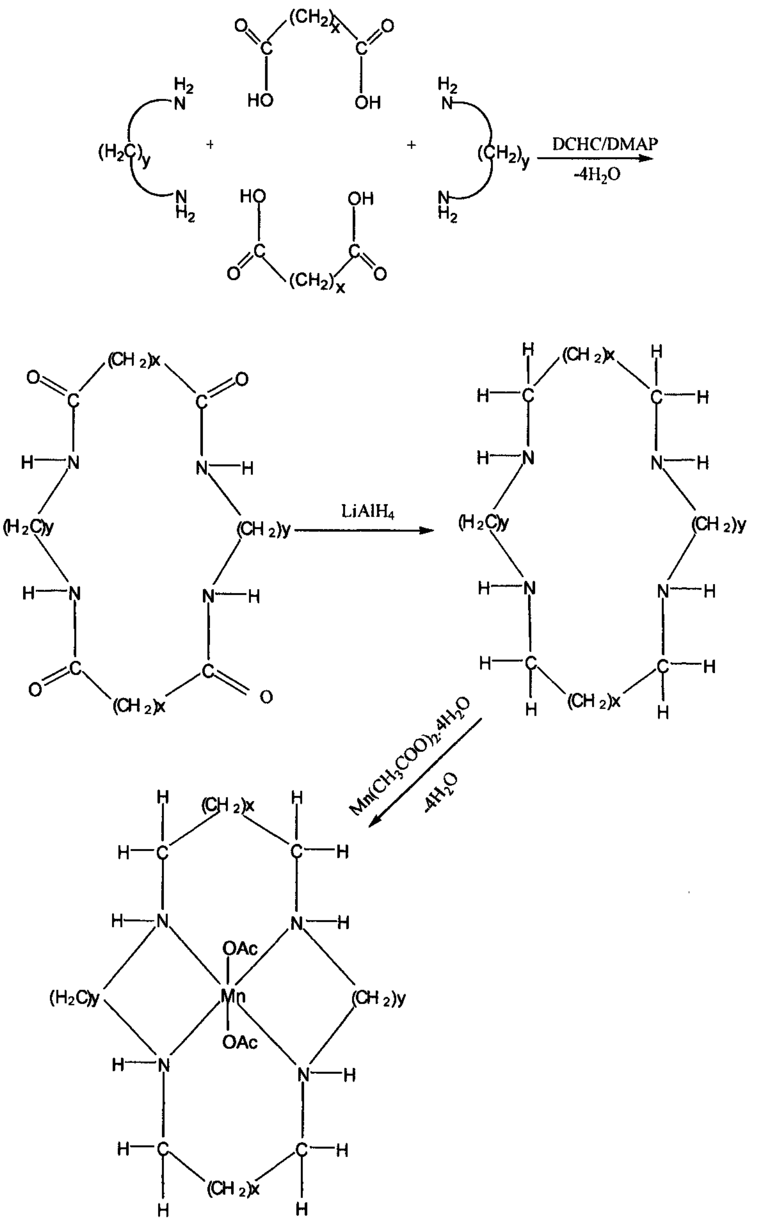

where:

| | | |
|---|---|---|
| $N_4TTD^1$ | x = 1 | y = 2 |
| $N_4TTD^2$ | x = 1 | y = 3 |
| $N_4TTD^3$ | x = 2 | y = 2 |
| $N_4TTD^4$ | x = 2 | y = 3 |
| $N_4TTD^5$ | x = 1 | y = 2 |
| $N_4TTD^6$ | x = 1 | y = 3 |
| $N_4TTD^7$ | x = 2 | y = 2 |
| $N_4TTD^8$ | x = 2 | y = 3 |

**Fig.2 Synthetic Routes of the Ligands and Complexes**

### Antimicrobial Assay :

The antifungal activities were evaluated against *Collectatrichum capsici, Penicillium notatum* and *Sceleratium rolfsii* by the Radial Growth Method /21/ using Czapek's agar medium. The compounds were dissolved in 50, 100 and 200 ppm concentrations in methanol and then mixed with the medium. The linear growth of the fungus was determined by measuring the diameter of the colony after 96 hours. The percentage inhibition was calculated as 100 (dc-dt)/dc, where dc and dt are the diameters of the fungus colony in the control and test plates, respectively.

Bacterial activities were evaluated by the Inhibition Zone Technique /23/. The organism used were *Escherichia coli* (-), *Staphylococcus aureous* (+) and *Klebsiella aerogenous* (-). The nutrient agar medium (Peptone, Beef extract, NaCl and Agar-Agar and 5 mm diameter paper discs, (Whatman No. 1) filter paper were used. The compounds were dissolved in methanol in 500 and 1000 ppm concentrations. The filter paper discs were soaked in these solutions of the compounds, dried and then placed in the petriplates previously seeded with the test organism. The petridishes were stored in an incubator at 30 ± 1°C for 24 hours. The zone of inhibition thus formed around each disc containing the test compound was measured accurately.

### *Mode ofAction :*

Potato dextrose media (PDA) rich in carbohydrates as the nutrient source is utilized by the microbes with the help of various enzymes. Metal based fungicides inhibit a wide range of enzymes involved in various metabolic pathways and ultimately causing the cell death. Early work on the mode of action of the fungicides showed that these compounds inhibit cell division. It was later shown that the specific site of the action is β-tubuline, a polymeric protein found in microtubules-essential component of the cytoskeleton. Phenyl and amine groups in the complexes effect nucleic acid synthesis and mitochondrial electron transport also. One may then expect at least the following regulatory processes to be operative /24,25/.

### *(i) Carbon Catabolic Regulation*

During the period of the rapid utilization of the carbon source, rapid utilization of the glucose or sucrose in the secondary metabolic pathways leading to toxins would be repressed or the activity of these pathways would be inhibited.

### *(ii) Nitrogen Catabolic Repression*

Excessive levels of rapidly assimilated forms of nitrogen (e.g. ammonium ion) could repress the formation of the enzymes concerned with the nitrogen transformation of the toxins intermediates.

### *(iii) Feed Back Regulation*

As toxins accumulate they would, in some instances, limit their own biosynthesis by inhibiting the activity of one or more enzymes earlier in their synthetic pathways.

***(iv)Feed Back Regulation by Primary Precursors***

Primary metabolites that are precursors of toxins could not act similarly by inhibiting the enzymes in the primary pathways prior to where they branch off into secondary ones.

***(v) Energy Charge Regulation***

High phosphate levels could reduce the availability of high energy phosphate (i.e. ATP and ADP). This would effectively inhibit a number of key reactions in primary metabolism, which, in turn, would cause a reduction in the activity of the secondary pathways liked to the toxin production.

***(vi)Induction***

The addition of certain primary metabolites (termed effectors) could induce the formation of enzymes in the secondary pathways leading to the toxin production. This effect would be aside from any function the effectors might have as precursors of the toxins.

For these organisms, even at low concentrations the inhibition of the growth of the micro-organism was found to be dependent on the concentration of the compounds. The results of biocidal activity have been compared with the conventional fungicide, ***Bavistin*** and the conventional bactericide *Streptomycin* used as standards. The results achieved from these studies have been listed in Tables 5 and 6, in which the antifungal activity indicated that the complexes are more active than the ligands. The variation in the effectiveness of the different biocidal agents against different organisms /26/ depends on the impermeability of the cell. The hydrocarbon tail functions as a lipophilic group /26/ to drive the compound through the semipermeable membrane of the cell.

The striking feature seen in the bacterial activity is the remarkable potential of the toxicity, for the gram (+) stain as compared to the gram (-) stain. The reason is the difference in the structure of the cell walls. The walls of the gram (-) cells are more complexes than those of the gram (+) cells. Over all, the results were appreciable when compared with a standard. The bioactivity increased on undergoing complexation but did not reach the efficacy of the standard at lower concentration. However, at higher ppm concentration the results achieved were satisfactory. The aforesaid studies are clearly worthy of further investigation.

***Percent Disease Control***

In this method compounds were tested in the field for controlling the disease caused by the causal organism. Two concentrations, 100 and 200 ppm, were used in different plots and observations were recorded.

$$\text{Percent Disease Incidence (PDI)} = \frac{\text{No. of infected plants}}{\text{Total no. of plants observed}} \times 100$$

$$\text{Percent Disease Control} = \frac{\text{PDI in treated plant} - \text{PDI in untreated plants}}{\text{PDI in untreated plants}} \times 100$$

**Table 5**

Fungicidal Screening Data of Tetraamide Ligands and the their Manganese Complexes.

| Compound | % Inhibition after 96 hours (Conc. in ppm) | | | | | | | | |
|---|---|---|---|---|---|---|---|---|---|
| | *Collectarichium capsici* | | | *Penicillium notatum* | | | *Sceleratium rolfsii* | | |
| | 50 | 100 | 200 | 50 | 100 | 200 | 50 | 100 | 200 |
| Bavistin standard | 90 | 100 | 100 | 88 | 100 | 100 | 87 | 100 | 100 |
| $N_4TTD^1$ | 39 | 61 | 80 | 48 | 73 | 86 | 38 | 61 | 78 |
| $N_4TTD^2$ | 47 | 65 | 85 | 40 | 66 | 81 | 47 | 62 | 81 |
| $N_4TTD^3$ | 34 | 63 | 72 | 31 | 59 | 73 | 31 | 57 | 70 |
| $N_4TTD^4$ | 37 | 44 | 53 | 34 | 47 | 55 | - | - | - |
| $TTD^5$ | 50 | 53 | 60 | 52 | 54 | 64 | - | - | - |
| $N_4TTD^6$ | 50 | 69 | 79 | 61 | 68 | 80 | 58 | 66 | 78 |
| $N_4TTD^7$ | 58 | 66 | 82 | 58 | - | - | 60 | 64 | . |
| $N_4TTD^8$ | 64 | 76 | 86 | 74 | 81 | 87 | 72 | 79 | 86 |
| $[Mn(CH_3COO)_2 (N_4TTD^5)]$ | 78 | 90 | 95 | 87 | 95 | 98 | 85 | 94 | 98 |
| $[Mn(CH_3COO)_2 (N_4TTD^6)]$ | 74 | 83 | 92 | 71 | 78 | 90 | 77 | 87 | 94 |
| $[Mn(CH_3COO)_2 (N_4TTD^7)]$ | 80 | 90 | 97 | 82 | 91 | 100 | 81 | 94 | 100 |
| $[Mn(CH_3COO)_2 (N_4TTD^8)]$ | 85 | 96 | 100 | 85 | 96 | 100 | 82 | 95 | 100 |

The fungus *Alternaria alternata* and brinjal plants were used for this purpose. The efficacy of tetraamidemacrocyclic ligands and their manganese complexes under *in vitro* condition and in the field condition was studied. The field experiments were laid out in randomized block design with three replications. The brinjal plants were raised in each plot. Compounds with a standard fungicide, ***Bavistin*** was tried in addition to check water spray.

Thirty days after sowing, the plants were inoculated artificially by spraying the conidial suspension. The conidial was prepared by crushing infected leaves in water. The inoculation was done in the evening. The first spray of the respective fungicide was given when lesions were first seen and were repeated after 10 days. Disease intensity was recorded 10 days after the second spray. The data were analysed statistically and disease control (%) was worked out (Table 7).

**Table 6**

Antibacterial Screening Data of Tertraamide Ligands and their Manganese(II) Complexes.

| Compound | Inhibition after 24 hours (Conc. in ppm) | | | | | |
|---|---|---|---|---|---|---|
| | *Eschrichia coli (-)* | | *Staphylococcus aureus (+)* | | ***Klebsiella aerogenous*** | |
| | 500 | 1000 | 500 | 1000 | 500 | 1000 |
| Standard (Streptomycin) | 95 | 100 | 88 | 100 | 25 | 42 |
| $N_4TTD^1$ | 15 | 29 | 24 | 35 | 36 | 58 |
| $N_4TTD^2$ | 18 | 26 | - | 32 | 35 | 61 |
| $N_4TTD^3$ | 19 | 25 | 20 | 29 | - | 59 |
| $N_4TTD^4$ | 22 | 38 | 22 | 35 | 39 | 60 |
| $N_4TTD^5$ | 18 | 24 | - | - | 35 | 64 |
| $N_4TTD^6$ | 34 | 50 | 42 | 53 | 59 | 84 |
| $N_4TTD^7$ | 28 | 50 | 36 | 147 | 42 | 59 |
| $N_4TTD^8$ | 39 | 56 | 59 | 83 | 84 | 100 |
| $[Mn(CH_3COO)_2 (N_4TTD^5)]$ | 47 | 65 | 60 | 69 | 88 | 100 |
| $[Mn(CH_3COO)_2 (N_4TTD^6)]$ | 42 | 59 | 48 | 63 | 47 | 68 |
| $[Mn(CH_3COO)_2 (N_4TTD^7)]$ | 53 | 79 | 55 | 83 | 55 | 88 |
| $[Mn(CH_3COO)_2 (N_4TTD^8)]$ | 65 | 76 | 68 | 79 | 65 | 84 |

**Table 7**

Efficacy of Tetraamide Ligands and their Manganese(II) Complexes on Leaf Spot of Brinjal Plant Caused by *Alternaria alternata* by Percent Disease Incidence Method

| Compound | Concentration in ppm | Inhibition replicates (Out of 50) | | | % Disease Incidence | % Disease Control |
|---|---|---|---|---|---|---|
| | | $R_1$ | $R_2$ | $R_3$ | | |
| Bavistin 0.2% | - | 8 | 4 | 9 | 18.66 | 81.33 |
| Control water spray | - | 34 | 39 | 42 | 76.66 | 23.33 |
| $N_4TTD^1$ | 100 | 25 | 27 | 18 | 46.66 | 53.34 |
| | 200 | 20 | 21 | 25 | 41.99 | 56.01 |
| $N_4TTD^5$ | 100 | 21 | 22 | 18 | 40.66 | 59.34 |
| | 200 | 14 | 21 | 20 | 36.79 | 63.21 |
| $[Mn(CH_3COO)_2 (N_4TTD^5)]$ | 100 | 18 | 17 | 15 | 23.33 | 66.67 |
| | 200 | 12 | 14 | 15 | 27.38 | 72.62 |
| $N_4TTD^4$ | 100 | 25 | 24 | 20 | 46.00 | 54.00 |
| | 200 | 19 | 25 | 21 | 43.11 | 56.89 |
| $N_4TTD^8$ | 100 | 22 | 20 | 17 | 39.33 | 60.67 |
| | 200 | 20 | 21 | 13 | 36.03 | 63.97 |
| $[Mn(CH_3COO)_2 (N_4TTD^8)]$ | 100 | 14 | 16 | 19 | 32.66 | 63.34 |
| | 200 | 11 | 13 | 15 | 26.00 | 74 |
| C.D. at 5% | - | - | - | - | 2.56 | 4.21 |

***Antifertility Activity***

Healthy, adult male albino rats of the Sprague Dawley strain were used in the present investigations. The rats were divided into five groups containing seven animals each. The first group (A) served as vehicle (olive oil) treated control. In the group (B), ligand $N_4TTD^2$ and group (D), ligand $N_4TTD^4$ 25 mg $kg^{-1}$ body weight suspended in 0.2 mL olive oil and given orally for a period of 60 days. The animals of group (C) and (E) received the same dose of the compounds $[Mn(N_4TTD^2)CH_3COO)_2]$ and $[Mn(N_4TTD^4)CH_3COO)_2]$, respectively for the similar period. These animals were screened for fertility test and autopsied for determinatin of detailed biochemical studies. Reproductive organs were excised, blotted free of blood, weighed and were frozen for biochemical estimations. The sperm motility and density of cauda epididymal spermatozoa, total protein, sialic acid, fructose and acid phosphate were determined by the standard laboratory techniques.

All the values of the body weight, organ weights, sperm dynamics and biochemical estimations were averaged, standard error of mean values were calculated and student's 't' test was applied for standard comparison /27/.

***(a) Body and Organ Weights***

Administration of the complexes did not bring about any significant change in the body weights of the treated rats. The weights of testes, epididymis, seminal vesicle and ventral prostate were decreased significantly (Table 8) in all experimental groups when compared with vehicle treated controls.

***(b) Sperm Motility and Sperm Density***

A significant ($P < 0.001$) decline in the sperm motility, sperm density in testes and cauda epididymis was noticed in the rats treated with the ligands and their complexes (Table 9).

***(c) Biochemical Events Leading to Infertility***

***Protein***

Protein contents of testes, epididymis, ventral prostate and seminal vesicle were reduced after the treatment of ligands their complexes with rats (Table 10).

***Fructose***

Fructose content of seminal vesicle was decreased in ligands and their complexes (Table 10).

***Sialic Acid***

Sialic acid contents of the testes, epididymis and auxiliary glands (seminal vesicle and ventral prostate) were depleted significantly in all experimental groups (Table 11).

***Cholesterol***

A significant increase in testicular cholesterol ($P<0.05$) content was recorded in rats treated with ligands and their complexes (Table 11).

**Table 8**

Changes in the Body Weight and Organs Weights of Reproductive Organs after Treatment with Tertraamide Ligands and their Mn(II) Complexes.

| Group | Treatment | Body weight (g) | | Mg/100 g body weight | | | |
|---|---|---|---|---|---|---|---|
| | | Initial | Final | Testes | Epididymis | Seminal vesicle | Ventral prostate |
| A | Control | 190.0 ± 12.0 | 220.0 ± 9.50 | 1050.0 ± 70.5 | 400.0 ± 28.5 | 340.0 ± 27.8 | 250.75 ± 30.50 |
| B | $N_4TTD^2$ | 180.0 ± 18.0 | 215.0 ± 10.5$^c$ | 805.± 50.0$^a$ | 345 ± 20.5$^a$ | 300.0 ± 10.5$^a$ | 200.0 ± 10.7 |
| C | $[Mn(CH_3COO)_2(N_4TTD^2)]$ | 185.0 ± 15.0 | 210.0 ± 17.0$^c$ | 715.0 ± 20.0$^a$ | 281.75 ± 10.5 | 250.0 ± 17.3$^b$ | 155.0 ± 10.9$^a$ |
| D | $N_4TTD^4$ | 175.0 ± 15.0 | 21.0± 10.0$^b$ | 745.0 ± 15.0$^c$ | 275.60 ± 10.0 | 270.0 ± 15.3$^b$ | 150.0 ± 15.0$^a$ |
| E | $[Mn(CH_3COO)_2(N_4TTD^4)]$ | 175.0 ± 10.0 | 225.0 ± 15.0$^c$ | 780 ± 15.0$^b$ | 270.0 ± 20.0$^b$ | 280.0 ± 10.0$^b$ | 185.0 ± 15.3$^a$ |

Values means of ± SE of six determinations a = P ≤ 0.05

b = P ≤ 0.001

**Table 9**

Altered Sperm Dynamics and Fertility Test after Treatment with Tetraamide Ligands and their Mn(II) Complexes.

| Group | Treatment | Sperm density (million/ml) | | Sperm motility | Fertility Test (%) |
|---|---|---|---|---|---|
| | | Testes | Epididymis | Cauda epididymis | |
| A | Control | 1.75 ± 0.09 | 45.52 ± 1.5 | 72.0 ± 5.21 | 95 (+ ve) |
| B | $N_4TTD^2$ | 1.91 ± 0.10$^b$ | 38.0 ± 0.5$^a$ | 51.0 ± 3.7$^b$ | 70 (- ve) |
| C | $[Mn(CH_3COO)_2(N_4TTD^2)]$ | 0.85 ± 0.19$^b$ | 30.0 0.4$^b$ | 47.0 ± 3.8$^b$ | 80 (- ve) |
| D | $N_4TTD^4$ | 0.80 ± 0.15 | 25.0 0.5$^a$ | 42.0 ± 2.5$^b$ | 75 (- ve) |
| E | $[Mn(CH_3COO)_2(N_4TTD^4)]$ | 0.69 ± 0.15$^b$ | 25.0 ± 0.3 | 40.0 ± 3.8$^b$ | 90 (- ve) |

Values means of ± SE of six determinations a = P ≤ 0.05

b = P ≤ 0.001

**Table 10**

Effects of Tetraamide Ligands and their Manganese (II) Complexes on Various Biochemical Parameters (Total Protein and Fructose) of Reproductive Organs.

| Group | Treatment | Total protein (mg/g) | | | | Fructose (mg/g) |
|---|---|---|---|---|---|---|
| | | Testes | Epididymis | Seminal vesicle | Ventral prostate | |
| A | Control | 225.0 ± 17.0 | 205.0 ±19.3 | 250.0 ± 10.8 | 230.0 ± 20.5 | 450.0 ± 30.0 |
| B | $N_4TTD^2$ | 150.0 ± 13.0[a] | 17.00 ± 15.0[a] | 190.0 ± 11.0[a] | 185.0 ± 15.35[a] | 360.0 ± 40.0[a] |
| C | $[Mn(N_4TTD^2)\ CH_3COO)_2]$ | 130.0 ± 10.7[b] | 12.50 ± 15.4[b] | 139.0 ± 12.5[b] | 135.0 ± 17.7[b] | 310.0 ± 35.0[b] |
| D | $N_4TTD^4$ | 145.0 ± 10.5[b] | 155.0 ± 15.8[b] | 175.0 ± 10.2[b] | 180.0 ± 15.5[b] | 368.0 ± 30.0[b] |
| E | $[Mn(N_4TTD^4)\ CH_3COO)_2]$ | 111.4± 5.0[b] | 138.0 ± 10.3[b] | 150.0 ± 117.7[b] | 158.0 ± 200[b] | 330.0 ± 20.0[b] |

Values means of ± SE of six determinations a = P ≤ 0.05

b = P ≤ 0.001

**Table 11**

Effects of Tetraamide Ligands and their Manganese (II), Complexes on Various Biochemical Parameters (Sialic acid and Cholesterol) of Reproductive Organs.

| Group | Treatment | Sialic acid (mg/g) | | | | Total cholesterol (mg/g) |
|---|---|---|---|---|---|---|
| | | Testes | Epididymis | Seminal vesicle | Ventral prostate | |
| A | Control | 7.30 ± 0.9 | 6.30 ± 1.3 | 6.80 ± 1.3 | 6.90 ± 0.5 | 7.30 ± 0.52 |
| B | $N_4TTD^2$ | 5.80 ± 0.7[a] | 4.90 ± 1.3[b] | 5.0 ± 0.8[a] | 5.1 ± 0.3[a] | 8.10 ± 0.20[a] |
| C | $[Mn(CH_3COO)_2(N_4TTD^2)]$ | 3.90 ± 0.9[b] | 3.70 ± 0.1[b] | 3.80 ± 0.8[b] | 3.10 ± 0.1[b] | 8.90 ± 0.52[b] |
| D | $N_4TTD^4$ | 5.65 ± 0.5[b] | 4.70 ± 0.1[b] | 5.20 ± 0.1[b] | 5.15 ± 0.4[a] | 8.05 ± 0.43[b] |
| E | $[Mn(CH_3COO)_2(N_4TTD^4)]$ | 4.30 ± 0.8[b] | 4.30 ± 0.8[b] | 4.20 ± 0.9[b] | 4.28 ± 0.5[a] | 8.30 ± 0.59[b] |

Values means of ± SE of six determinations a = P ≤ 0.05

b = P ≤ 0.001

***Acid Phosphate***

Acid phosphate activities in testes, epididymis and ventral prostate of rats revealed a significant ($P < 0.05$) decrease, following the treatment of ligands and their Mn(II), complexes.

The present study evaluated the effects of tetraamide ligands and their Mn(II) complexes on reproductive functions of the rats. They caused a significant decrease in the sperm motility and density. Sperm motility and fertilizing capacity of spermatozoa were affected severely, which could be due to the androgen deficiency /28/.

The Mn(II) complexes caused reduction in the weight of the accessory sex organs which indicates the atrophy of glandular tissue and also reduction in the secretion ability, thus reflecting the decreased levels of **the** testosterone[29]. Decreased contents of the protein[30] in the treated rats could be due to the androgen deprivation. The sialic acid contents of the testes and accessory organs which were reduced in manganese complex treated rats also support the androgen depletion[31].

Present study suggests that the complex [$Mn(CH_3COO)_2(TTD^4)$] is more effective fertility inhibitor in male rats and it is due to the synergistic action of the Mn(II) moiety.

### ACKNOWLEDGEMENT

One of the authors (A.C.) is thankful to CSIR New Delhi for financial assistance in the form of RA vide no. 9/149 (374)/2K2, EMR-1.

### REFERENCES

1. D.F. Berger, E.C. Long, *Inorg. Chem.*, **31**, 262 (1992).
2. M. Hirai, K. Shinozuka, H. Sawai, S. Ogawa, *Chem. Lett.*, **10,** 2023 (1992).
3. E. Kimura, *Pure Appl. Chem.*, **65** (1993) 355
4. E. Kimura, M. Shinoya, A. Hoshino, T. Ikeda, Y. Yanada, *J. Am. Chem. Soc.*, **114,** 10134 (1992).
5. J.G. Muller, X. Chen, A.C. Dadiz, S.E. Rokita, C.S. Burrows, *Pure Appl. Chem.*, **65**, 545 (1993).
6. N. Fahmi, R.V.Singh, *Transition Met. Chem.*, **19**, 12 (1994).
7. N. Fahmi, S.C.S. Jadon, R.V. Singh, *Phosporous, Sulfur Silicon*, **81** (1993) 133.
8. T.J. Collins, R.D. Powell, C. Slebocknick, E.S. Uffelman, *J. Am. Chem. Soc.*, **133,** 8419 (1991).
9. C. Saxena, S.C. Joshi, R.V. Singh, *Bull. Chem. Soc. Jpn.*, **67**, 1007 (1994).
10. D. Singh R.B. Goyal, R.V. Singh, *Appl. Organomet. Chem.*, **5**, 45 (1991).
11. V. Mckee, Adv. *Inorg. Chem.* **32**, 323 (1993).
12. T.J. Collins, K.L. Kostka, E.S. Uffelman, T.L. Weinberger, *Inorg. Chem.*, **30**, 4204 (1991).
13. Y.A. Ibrahim, A.H.M. Elwaky, G.M.M. Elkaresh, *J. Chem. Res.*, **5**, 414 (1994).
14. D. Parker, *Chem. Soc. Rev.* **19,** 271 (1990).
15. R.H. Dixon, *Reproductive Toxicology,* Raven Press, New York. 1985; p.309.
16. P. Schulfer, H. Oyanguron, V. Maturana, A. Valenzuela, E. Cruz, V. Plaza, E. Schmidt, R. Hadded, *Industrial Medicine and Surgery,* **26,** 165 (1957).
17. A. Chaudhary, S. Dave, R.K. Saini, R.V. Singh, *Main Group Met. Chem.*, **24**, 217 (2001).
18. N.B. Colthup, L.H. Dally and S.E. Wiberly, *Introduction of Infrared and Raman Spectroscopy,* Academic Press, New Delhi, 1964.
19. R.K. Agarwal, S.K. Gupta, *Reo Roum Chem.*, **32,** 447 (1987).
20. M.B.H. Howlader, M.S. Islam, M.R. Karim, *Indian J. Chem.*, **39A,** 407 (2000).
21. R.S. Lal, A. Kumar, J. Chakraborty, *Indian J. Chem.*, **40A,** 422 (2001).
22. A. Bansal, R.D. Singh, R.V. Singh, *Metal Based Drugs*, **7,** 211 (2000).

23. K. Sharma, S.C. Joshi, R.V. Singh, *Metal Based Drugs* **7,** 237 (2000).
24. E.C. Moore, M.s. Zeleck, K.C. Agarwal, A.L. Sartorelli, *Biochemistry,* 14492 (1970).
25. P.G. Lawrence, P.L. Harold, O.G. Fancis, *Antibiotic, Chemotherapy,* **5,** 10134 (1980).
26. S. Belwal, R.K. Saini, R.V. Singh, *Indian J. Chem.,* **37A,** 245 (1998).
27. J. Ipstein, F. Poly, In: *Benchroft's Introduction to Biostatistic,s* 2nd ed. (Harper International), 1970; p.44.
28. G. Gupta, A.K. Srivastava, B.S. Shetty, *Indian J. Exptl. Biol.,* **31,** 305 (1993).
29. D. Malarvizhi, P.P. Mathu, *Indian J. Exptl. Biol.,* **33,** 281 (1995).
30. G.S. Prins, L. Birch, *Endrocrinal,* **130,** 169 (1993).
31. B.S. Setty, S.S. Riar, A.K. Kar, *Fertil. Stenl.,* **28,** 674 (1979).

